# Genetic diversity and selection signatures in a gene bank panel of maize inbred lines from Southeast Europe compared with two West European panels

**DOI:** 10.1186/s12870-023-04336-2

**Published:** 2023-06-14

**Authors:** Vlatko Galić, Violeta Anđelković, Natalija Kravić, Nikola Grčić, Tatjana Ledenčan, Antun Jambrović, Zvonimir Zdunić, Stéphane Nicolas, Alain Charcosset, Zlatko Šatović, Domagoj Šimić

**Affiliations:** 1 Agricultural Institute Osijek, Južno predgrađe 17, Osijek, HR31000 Croatia; 2 Centre of Excellence for Biodiversity and Molecular Plant Breeding (CroP-BioDiv), Svetošimunska cesta 25, Zagreb, HR10000 Croatia; 3 Maize Research Institute Zemun Polje, Slobodana Bajića 1, Belgrade, 11185 Serbia; 4 GQE ‑ Le Moulon, INRAE, Univ. Paris‑Sud, CNRS, AgroParisTech, Université Paris-Saclay, Gif‑sur‑Yvette, 91190 France; 5 Faculty of Agriculture, University of Zagreb, Svetošimunska cesta 25, Zagreb, HR10000 Croatia

**Keywords:** Maize, Selective sweep, Diversity, Gene ontology, Genetic resources

## Abstract

Southeast Europe (SEE) is a very important maize-growing region, comparable to the Corn belt region of the United States, with similar dent germplasm (dent by dent hybrids). Historically, this region has undergone several genetic material swaps, following the trends in the US, with one of the most significant swaps related to US aid programs after WWII. The imported accessions used to make double-cross hybrids were also mixed with previously adapted germplasm originating from several more distant OPVs, supporting the transition to single cross-breeding. Many of these materials were deposited at the Maize Gene Bank of the Maize Research Institute Zemun Polje (MRIZP) between the 1960s and 1980s. A part of this Gene Bank (572 inbreds) was genotyped with Affymetrix Axiom Maize Genotyping Array with 616,201 polymorphic variants. Data were merged with two other genotyping datasets with mostly European flint (TUM dataset) and dent (DROPS dataset) germplasm. The final pan-European dataset consisted of 974 inbreds and 460,243 markers. Admixture analysis showed seven ancestral populations representing European flint, B73/B14, Lancaster, B37, Wf9/Oh07, A374, and Iodent pools. Subpanel of inbreds with SEE origin showed a lack of Iodent germplasm, marking its historical context. Several signatures of selection were identified at chromosomes 1, 3, 6, 7, 8, 9, and 10. The regions under selection were mined for protein-coding genes and were used for gene ontology (GO) analysis, showing a highly significant overrepresentation of genes involved in response to stress. Our results suggest the accumulation of favorable allelic diversity, especially in the context of changing climate in the genetic resources of SEE.

**Supplementary Information:**

The online version contains supplementary material available at 10.1186/s12870-023-04336-2.

## Key message

Analysis of diversity and selection in a Southeast European panel of maize inbred lines showed historical material swap accompanied by selection for adaptation to Green-Revolution agronomic practices.

**Supplementary Information:**

The online version contains supplementary material available at 10.1186/s12870-023-04336-2.

## Introduction

South Eastern Europe (SEE), including the agriculturally important Pannonian plain, can be considered a European counterpart to the US Corn Belt with well-adapted temperate dent germplasm and more than 20% of the crop areas sown with maize [1]. Moreover, more than 35% of the European grain maize was produced in Serbia, Romania and Hungary, and continental Croatia in the period from 2010–2014 [2]. In more recent reports, Croatia, Serbia, Romania, and Hungary, in 2018 and 2019, together contributed 52% and 51%, respectively of the European Union + Serbia’s total maize grain production [3, 4].

Unlike in West Europe, data about the molecular diversity of maize genetic material in SEE is limited [5–7] and no comprehensive genotyping data set is available. Nonetheless, utilization of the SEE maize for its favorable alleles and diversity has been long speculated [8], with most of the materials deposited in gene banks. One such bank is Maize Research Institute Zemun Polje (MRIZP) Gene Bank conserving > 6000 accessions, of which > 2000 are local landraces collected throughout the former Yugoslavia and > 4000 accessions are inbred lines and landraces originating from 40 different countries [9], representing one of the largest maize collections in the world [10]. The relevance of the plant genetic resources in the context of breeding has at least two converging aspects. First is the conservation of the biodiversity that has been narrowed by the way the historical diversity has been utilized [11]. Second is the mining and utilization of favorable variability using all available modern breeding tools, such as dense genotyping, high throughput phenotyping, etc. to overcome issues such as linkage drag [12–16].

Aside from West Europe, where the heterotic groups used for breeding are European flint and North American dent, the main heterotic groups in SEE are placed within North American dent heterotic patterns like in North America [17]. After World War II, some of the European traditional varieties were used to develop lines adapted to European conditions [18, 19]. These were crossed with materials imported from the US, including hybrids such as WF9 x Hy, Hy x Oh07, W32 x W187, etc. during the 1950s [20, 21]. Growing the locally adapted maize cultivars, developed from the European traditional varieties was so popular in the SEE during the 1960s that it was even speculated to surpass the production of the US hybrids in the following decades [8], mostly due to higher expenses of seed production. However, the performance improvement necessitated seed producers to accept the novel methods such as hybrid breeding, and the first original-pedigree double-cross hybrids were released as soon as the beginning of the 1960s [18], followed by three-way and single cross hybrids. The source of that-time modern introduced US germplasm was the organized unlimited production of US double-crosses, open-pedigree hybrids in Yugoslavian public research institutes as part of the American Aid plan through the Foreign Organization Administration [22]. The imported inbreds in the mid-1950s were: Wf9, 38-11, Hy, L317, N6, K148, K150, M14, W32, W187, A374, A375, and Oh07.

Although in modern maize breeding programs, considerable amount of genetics variation is attributable to some of these inbreds [23, 24], modern maize breeding usually follows heterotic patterns as described by [25–27]. Briefly, single crosses are created from inbreds belonging to different heterotic pools which strain from several historic US breeding programs [27], with highest leverage of Reid [28]. Modern commercial maize hybrids grown around the world today are mostly single crosses developed through tangled crossing and testing schemes in target populations of environments by multi-national companies [29]. Only marginal market shares are held by small companies and public institutions, assumingly subjecting the resilience of seed production to changes in ownership or market conditions. Furthermore, there is a concern in the breeding community that advanced-cycle pedigree breeding schemes in maize might lead to the available germplasm becoming more genetically narrow [28, 30]. Due to the structured heterotic patterns in maize breeding, population-level diversity is maintained, at least between the heterotic groups. However, to sustain the long-term breeding progress, exploiting the new germplasm resources is inevitable, especially for adaptation traits [31–33]. Broadening of the genetic diversity by germplasm introductions often results in signs of sudden population expansions. Studying the selection during a certain historical period in historical accessions relies on these processes, although bottlenecks and significant drifts are also present in modern maize breeding germplasm [23]. Most often, certain genomic regions show signs of reduced variability seen as deviations of site frequencies from the assumed neutral model [34]. These deviations are termed soft sweeps, as distinct patterns of variability reduction are produced in the proximity of the selected site [35]. Unlike soft sweeps, in hard sweeps, favorable haplotypes rise to high frequencies in short times, severely reducing the variability of linked sites.

The aims of this study were to screen a gene bank panel of densely genotyped maize inbred lines from Southeast Europe for diversity and selection, and to compare it with other two European germplasm panels. Furthermore, the genomewide selection indices were used to mine available databases for genes used to carry out the gene ontology (GO) enrichment analysis, providing an overview of historical selection for adaptation to the Southeast European climate.

## Material and methods

### Plant material

A subset of 572 inbred accessions of the Maize Gene Bank of the Maize Research Institute Zemun Polje (MRIZP) was used to carry out this study (hereby referred to as MRIZP panel). Accessions i.e. inbred lines were chosen in a way to represent the diversity of introduced or de-novo developed material from the SEE breeding programs, along with several inbreds with collection attributes from other countries. In the MRIZP panel, there were 220 accessions collected from Bulgaria, 132 from ex-Yugoslavia, 54 from Romania, 42 from Hungary,18 from ex-Czechoslovakia, 13 from Poland and 7 from Greece. In addition, the MRIZP panel contained inbreds that did not originate from SEE: 47 from ex-USSR, 12 from USA, 8 from Mexico, 7 from Iran, 3 from France, 2 from both Canada and ex-East Germany, and 1 from each of ex-People’s Republic of Korea, Pakistan, Switzerland, Argentina and unknown origin. For further analyzes, the subpanel MRIZP-SEE was created, carrying only inbreds from Southeast Europe (former Yugoslavia, Bulgaria, Romania, and Greece) and the bordering Pannonian plain (Hungary). All additional information about the used inbred lines and subpanel designations is available in Supplementary Table S1.

### Genotyping and data management

The MRIZP panel was genotyped with the Axiom™ 600 k Maize SNP Genotyping Array with 616,201 variants, of which 6,759 represent insertions/deletions [14, 36]. All steps of the DNA analysis were conducted by SGS TraitGenetics GmbH, Germany, including standard protocols for DNA extraction and marker quality control. Two other publicly available genotypic matrices anchored with the same genotyping array were used inthis study. The first data was from [14, 36], on 155 elite dent or European flint / Northern flint inbred lines, mainly from German and French public breeding programs (TUM panel), and the second was the data from [37], on 247 dent inbred lines (DROPS panel), described in detail by [38]. Both data sets contained inbreds developed in Europe along with the most important US inbreds with expired Plant Variety Protection (ex-PVP) and public inbreds. Additional information about the inbred lines from TUM and DROPS datasets is available in Supplementary Table S1.

The data from all three datasets were merged using a custom R script, and insertions/deletions were removed, leaving 500,167 markers. Markers were further filtered to remove excessive heterozygotes (2.5%) and missing data (5%) in TASSEL software [39] version 5.2.64, leaving a final set of 460,243 filtered markers. The missing data were imputed using the LinkImpute method [40] with 50 sites in high linkage disequilibrium and 30 nearest neighbors. Overall mean heterozygosity proportion was low in unimputed data (0.0175) as well as in the imputed data (0.0181). Before imputation, proportion of missing data was 0.00838, and imputation resulted in lowering this proportion to 0.0003. Supplementary table S1 shows full line-level genome summaries for imputed and unimputed data. For population structure analysis, all markers were thinned to 1000 base pair distance, leaving 166,755 sites.

### Population structure

Population structure was determined by combining two methods in two different datasets. Datasets were: Southeastern Europe dataset with 455 inbreds with collection attributes in MRIZP database from Southeastern Europe, and a full dataset of 974 lines including the 572 inbreds in the MRIZP panel, the 155 inbreds of TUM panel and the 247 inbreds from DROPS panel. TUM and DROPS panels were subject to the same filtering and quality checking and imputation procedures as the MRIZP dataset. Principal coordinate analysis (multi-dimensional scaling, PcoA) was performed with 20 components assumed, with a thinned marker set using an identity-by-state distance matrix between 166,755 sites as input in TASSEL software version 5.2.64. The number of components for interpretation was chosen based on the presence of an “elbow” in the plot of eigenvalues, which appeared at three components in the analysis of a full dataset of 974 inbred lines and two components in the analysis of inbreds from the MRIZP-SEE subpanel.

To correctly infer the underlying genetic structure of the assessed germplasm, Admixture analysis [41] was performed with 166,755 thinned and imputed sites. Based on the findings of Puechmaille [42] that uneven sampling of subpopulations leads to underestimates of the true number of K, parameters MedMed K, MedMeanK, MaxMed K, and MaxMean K were calculated using the StructureSelector software [43]. All parameters converged at K = 7 for the full dataset of 974 inbreds, while non-zero values varied for MedMean K and MaxMean K between K = 2 and K = 3 in the MRIZP-SEE subpanel. All inbreds with Q > 0.7 to any of the inferred groups were considered members of the associated group, while inbreds with Q < 0.7 were considered admixed.

### Signatures of selection and candidate genes

The creation of genotyping arrays is based on genotyping the genetic materials with already discovered polymorphisms [44]. This approach can generate considerable ascertainment bias, mostly seen through spurious minor allele frequency (MAF) distribution across loci and low SNP call rates for some accessions, which is due to the limited diversity used for array formation. In our research, the SNP call rate was > 0.985 and MAF was > 0.1 for all used markers. Despite the high quality of the generated genotype data, the efficient strategy proposed by Malomane et al. [45] to mitigate the ascertainment bias was applied. Namely, stringent LD-based SNP filtering was carried out using the Plink 1.9 software [46] with flag *indep 50 5 2*, representing a moving window variance inflation factor (VIF) based SNP pruning within 50 SNP windows and a moving step of 5, where VIF is calculated as $VIF=1/(1-{r}^{2})$. Pruning left 58,264 markers in the dataset.

The fixation index (F_ST_) [47] screening was estimated using VCFtools software version v0.1.16 by using the --weir-fst-pop flag and a window size of 50 SNP markers. The MRIZP-SEE subpanel was set as one population, while the remaining 519 inbreds from all three panels were treated as a second (contrast) population. Further, the markers that crossed an F_ST_ threshold of 0.153431 were analyzed for extended haplotype homozygosity per site (EHHS) in R package *rehh* version 3.2.2 [48]. The F_ST_ threshold of 0.153431 was determined at a $\alpha <0.001$ cutoff level.

Secondly, in the MRIZP-SEE subpanel, the scan for genetic hitchhiking was carried out using the Sweep Detector (SweeD) software [49]. SweeD is an implementation of a likelihood-based sweep detection method proposed by Nielsen et al. [34] optimized for large SNP matrices and parallel computing. The method uses the likelihood of a neutral model calculated and based on all SNP markers as the denominator and the likelihood of selection for a certain genomic location as a numerator to compute the composite likelihood ratio (CLR) statistics. SweeD software version 3.3.2. was run in Linux OS with a grid size of 10,000 positions per chromosome for the calculation of CLR statistics. CLR threshold of 6.530486 was determined from the top 100 hits, representing a cutoff threshold of $\alpha <0.001$. Start and end markers of F_ST_ and CLR significant hits were converted from AGPv2 to B73_RefGen_v4 assembly in the in Ensembl Plants [50] tool Assembly Converter (https://plants.ensembl.org/Zea_mays/Tools/AssemblyConverter, accessed: 4^th^ January 2023). Overlapping regions were also tested for extended haplotype homozygosity score (EHHS) in R/*rehh* library [48]. Extended haplotype homozygosity score relies on reduction of genetic variation in the broader haplotypic regions over population of sequences. Since the EHHS robustness in selfing species is subject of independent research [51], the score was used in this research only as a confirmation of selection within genomic regions under selection detected by F_ST_ and CLR. The converted markers were used to mine the EnsemblPlants Genes database, release 51 via the BioMart tool (https://plants.ensembl.org/biomart/martview, accessed: 4^th^ January 2023) for genes within the detected genomic regions. The mined genes from BioMart analysis were used as input for gene ontology (GO) analysis in Protein Analysis Through Evolutionary Relationships (PANTHER) Classification System (http://pantherdb.org/about.jsp, accessed: 4^th^ January 2023) [52]. The GO terms *Molecular function*, *Biological process*, and *Cellular function* were analyzed by means of a statistical overrepresentation test, and the *p*-values were corrected according to Bonferroni correction for multiple testing, as the same genes can be involved in multiple processes.

## Results

### Panel composition and population structure

The MRIZP panel consisting of 572 inbred lines segregated at 99.99% of filtered and imputed markers with an average MAF of 0.255 (Table 1).

**Table 1:** Summary of genotypic data for the MRIZP maize panel and MRIZP-SEE subpanel as well as publicly available genotypic data for the two West European panels of DROPS [37] and TUM [14]

Panel	Number of inbreds	Number of sites (all panels)	Segregating sites	Average MAF
MRIZP	572	460,263	460,241	0.255
MRZIP-SEE	455		460,241	0.249
DROPS	247		460,242	0.245
TUM	155		460,239	0.264
Total	974		460,243	0.255

**Fig. 1:**
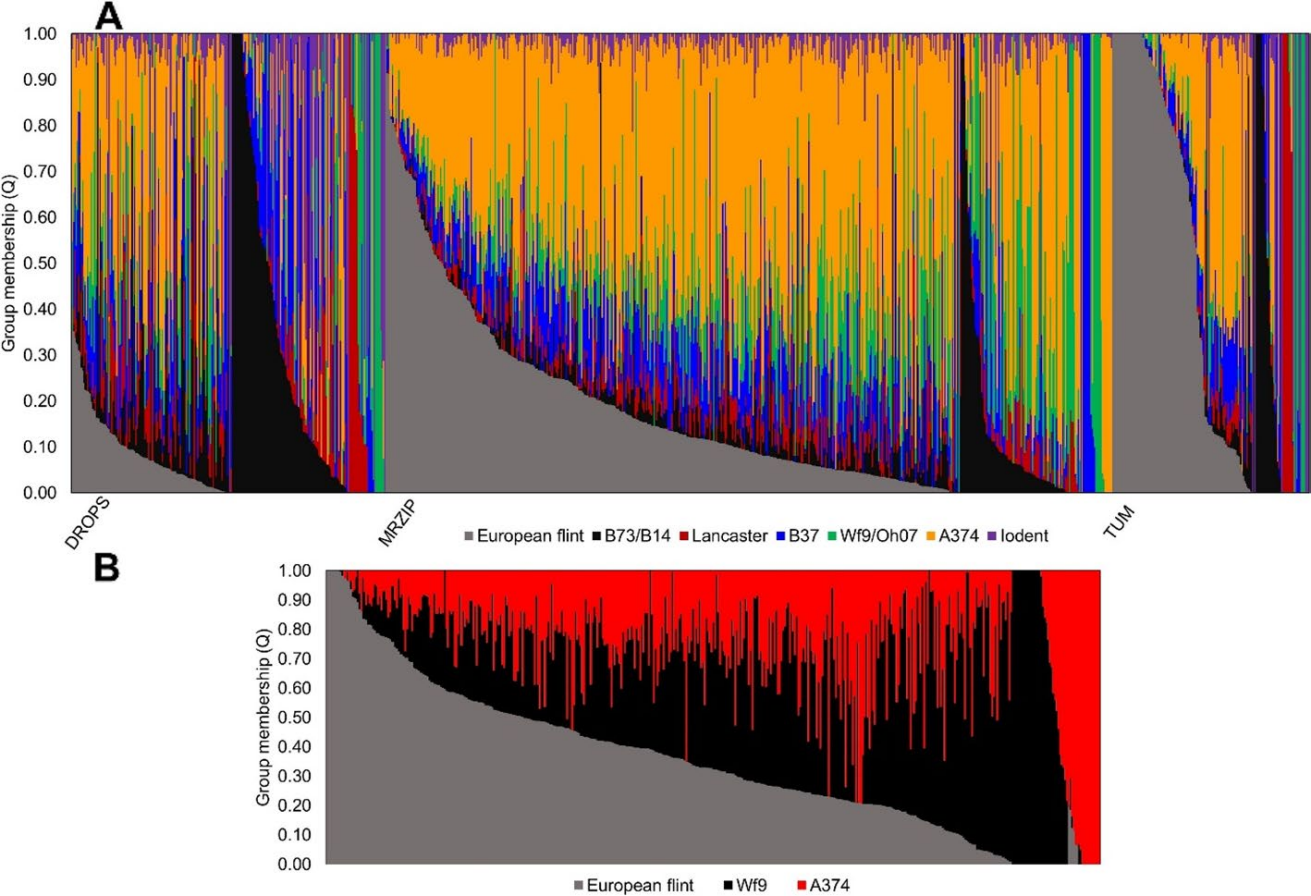
Group membership coefficients (Q) from Admixture analysis for K = 7 in 974 inbreds from three assessed panels: DROPS, MRIZP, and TUM (**A**), and group membership coefficients (Q) from admixture analysis for K = 3 in 455 inbreds from the MRIZPSEE sub panel (**B**). Each ancestral population has been designated by commonly known representatives or groups, K1 being designated European flint, K2 B73/B14, K3 Lancaster, K4 B37, K5 Wf9/Oh07, K6 A374, and K7 Iodent (**A**), while (**B**) K1 was designated European flint, K2 Wf9, and K3 A374

All parameters of Structure Selector [43] converged at seven populations (K = 7) in the full dataset of 974 inbreds, while differences were observed between parameters for K between 2 and 3 in the MRIZP panel (Supplementary Figs. 1 and 2). Group memberships analysis showed that most inbreds from the MRIZP panel had admixed origin, with only 135 inbreds showing membership coefficient (Q) > 0.7, 68 inbreds showing Q > 0.8 and 37 inbreds with Q > 0.9 in any of the determined groups (Supplementary table 1). The relative lack of an Iodent group was observed in the MRIZP panel, with closest grouping lines SEE-HMV107 and SEE-SD40 from Hungary and former Yugoslavia showing membership coefficients of 0.715 and 0.705 to the Iodent (K7) group. Interestingly, 47.9% of inbreds in the SEE panel showed a membership coefficient Q > 0.1 in the European flint group, but only sixteen inbreds showed Q > 0.7. The highest contributors to the European flint group with Q > 0.99 were two inbreds from the MRIZP panel (CK48_2 and FRC123), along with 24 inbreds from the TUM panel, namely: F283, F7012, F902, FC13, FC23, FV11, FV131, FV268, FV70, FV83, Fv230, Il14H, Ki11, Ky21, LH119, W117, X1G0896.DH116, X1G0896.DH123, X1G0896.DH212, X1G0897.DH102, X1G0897.DH135, X1G0897.DH203 (Supplementary Table S1). Further, 44.9% (257) inbreds showed Q > 0.1 in the B37 group, 38.9% (223) inbreds showed Q > 0.1 in the Wf9/Oh07 group, while 91.1% (521) inbreds showed Q > 0.1 in the A374 group (Fig. 1A). Thus, only a small number of inbreds from MRIZP retained clear membership in a single group.

Admixture analysis of the MRIZP-SEE subpanel alone (Fig. 1B) showed three ancestral groups (K = 3). The inbreds with Q > 0.7 in group K1 grouped most closely with the European flint material in K = 7 analysis (Fig. 1A) but with Q in the K = 7 analysis of no more than 0.72. The second group (K2) contained inbred lines with Q > 0.7 was grouped around Wf9 in the K = 7 analysis. Finally, inbreds in the K3 group were assigned to A374 pool in the K = 7 analysis. Inbreds with Q > 0.7 for K1 were collected from Bulgaria (29), former Yugoslavia (12), Greece (3), and Romania (4). Inbreds from K2 (Wf9) were also mostly collected from Bulgaria (29), Romania (6), Hungary (10) and former Yugoslavia (14), while in K3, most of the lines were collected from Bulgaria (14), former Yugoslavia (8), followed by Hungary (2) and Romania (2).

**Fig. 2:**
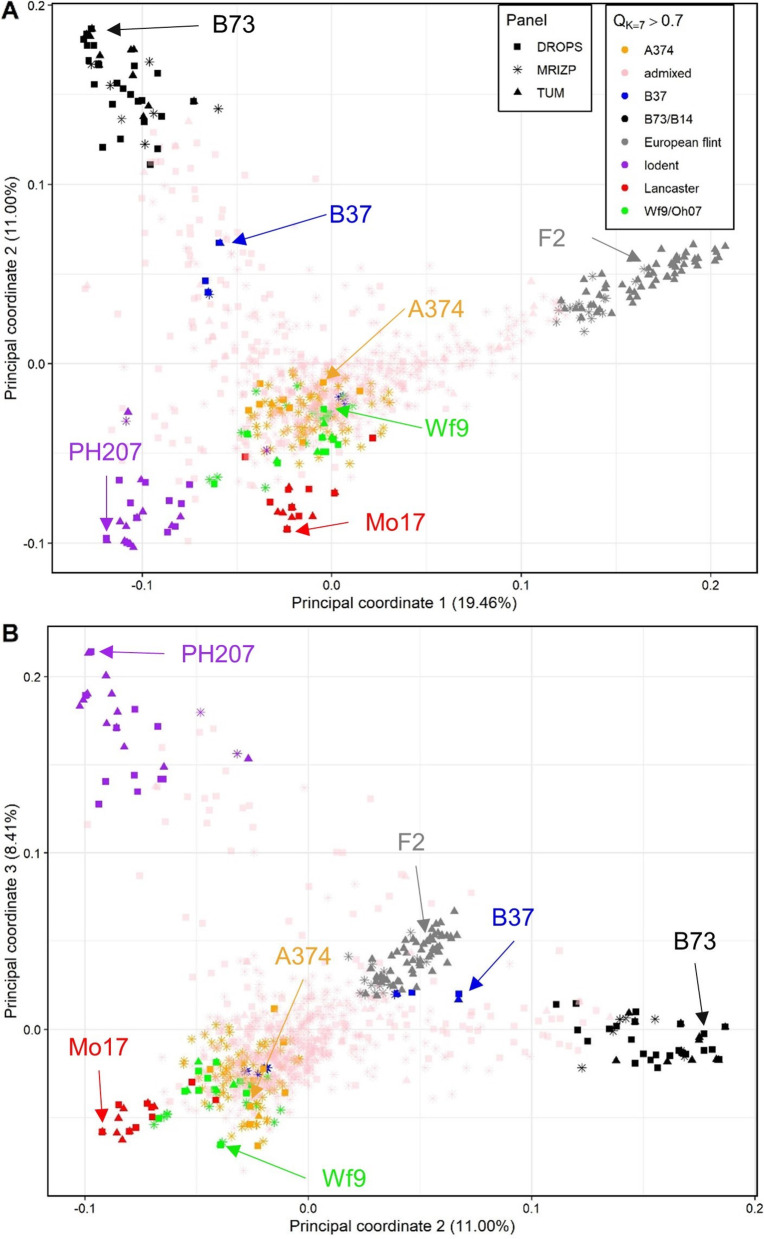
Results of principal coordinate analysis of 974 inbred lines from three genotyping panels (MRIZP, TUM and, DROPS) in coordinates: 1 vs. 2 (**A**) and coordinates 2 vs. 3 (**B**). Colored characters represent inbreds with Q > 0.7 for any of groups (K) in the Admixture analysis with K = 7 (Fig. 1A), while admixed individuals appear in pale pink. Founder lines are designated with names and arrows in color of their respective groups

The PcoA analysis of the full dataset (Fig. 2) showed groupings of inbreds assigned to different Ks in Admixture analysis. The most distinct groups in the 2D plane of the first two principal coordinates (Fig. 2A) were those assigned to European flint material (K1), Stiff Stalk Synthetic (K2), Lancaster (K3), and Iodent (K7). Inbreds from K4 to K6 grouped more closely, with inbreds from the K4 pool forming two distinct clusters in PCoA analysis, the upper subcluster representing original B37 inbred in panels DROPS and TUM, and other representing inbreds around French inbred F564 (Argentinian flint) and P352 (Supplementary table S1). The 2D projection plane with PCoA2 and PCoA3 (Fig. 2B) did not reveal any additional information regarding the grouping of inbreds.

**Fig. 3:**
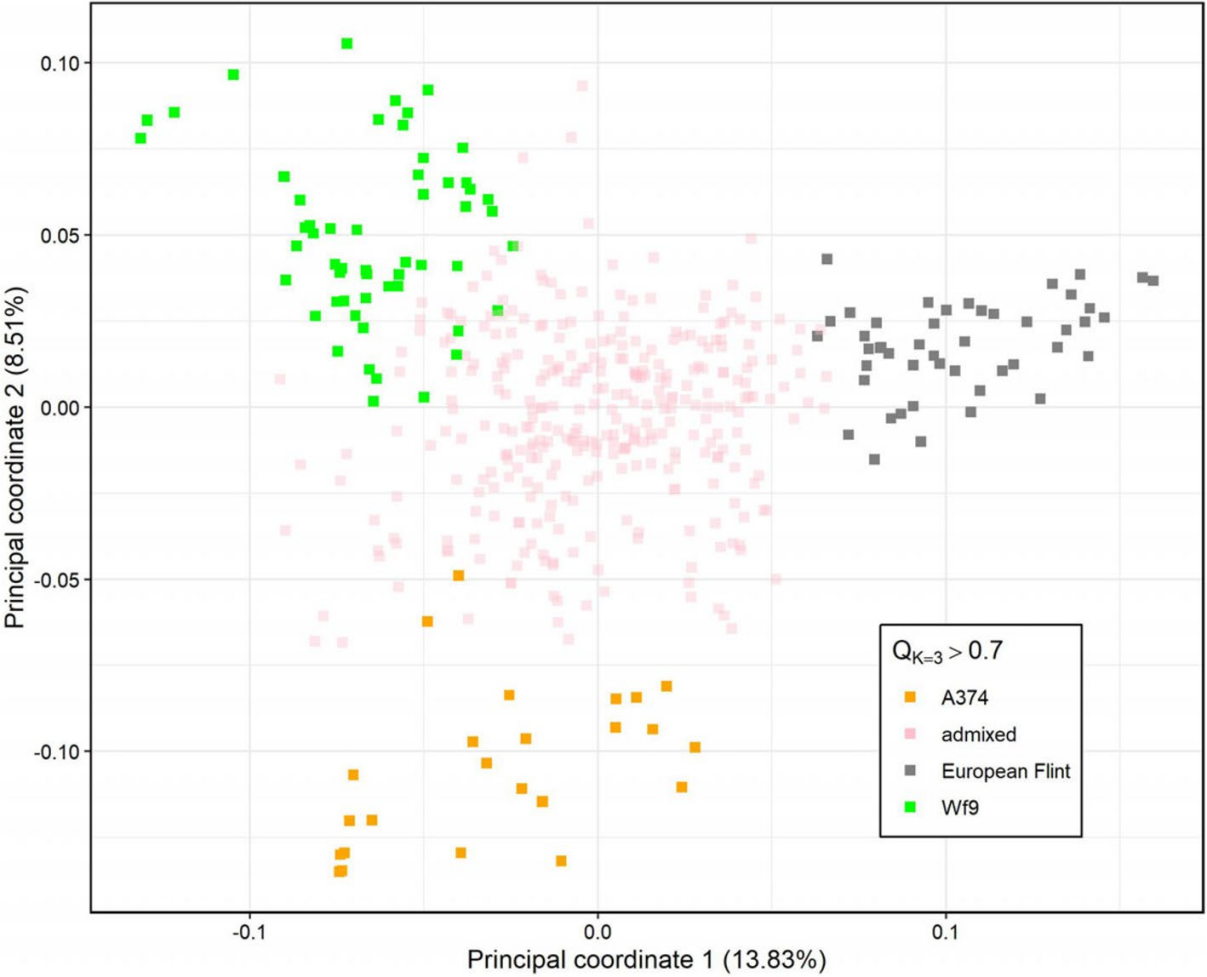
Results of principal coordinate analysis of 455 inbred lines from the MRIZP-SEE subpanel for coordinates: 1 vs. 2. Colored characters represent inbreds with Q > 0.7 for any of groups (K) in the Admixture analysis with K = 3 (Fig. 1B), while admixed individuals appear in pale pink. Additionally, the designations of inbreds with Q > 0.7 in K = 2 analysis are shown

The PcoA analysis of 455 inbreds from the MRIZP-SEE subpanel with collection-site attributes belonging to Southeast Europe showed distinct groupings of inbreds belonging to three populations (K) identified in the Admixture analysis (Fig. 3, additional info available in Supplementary table S2). Compared to the K = 2 analysis dividing the plane to left and right (data not shown), the K = 3 analysis showed a tripartite grouping (Fig. 3).

### Signatures of selection in maize from Southeast Europe

**Fig. 4:**
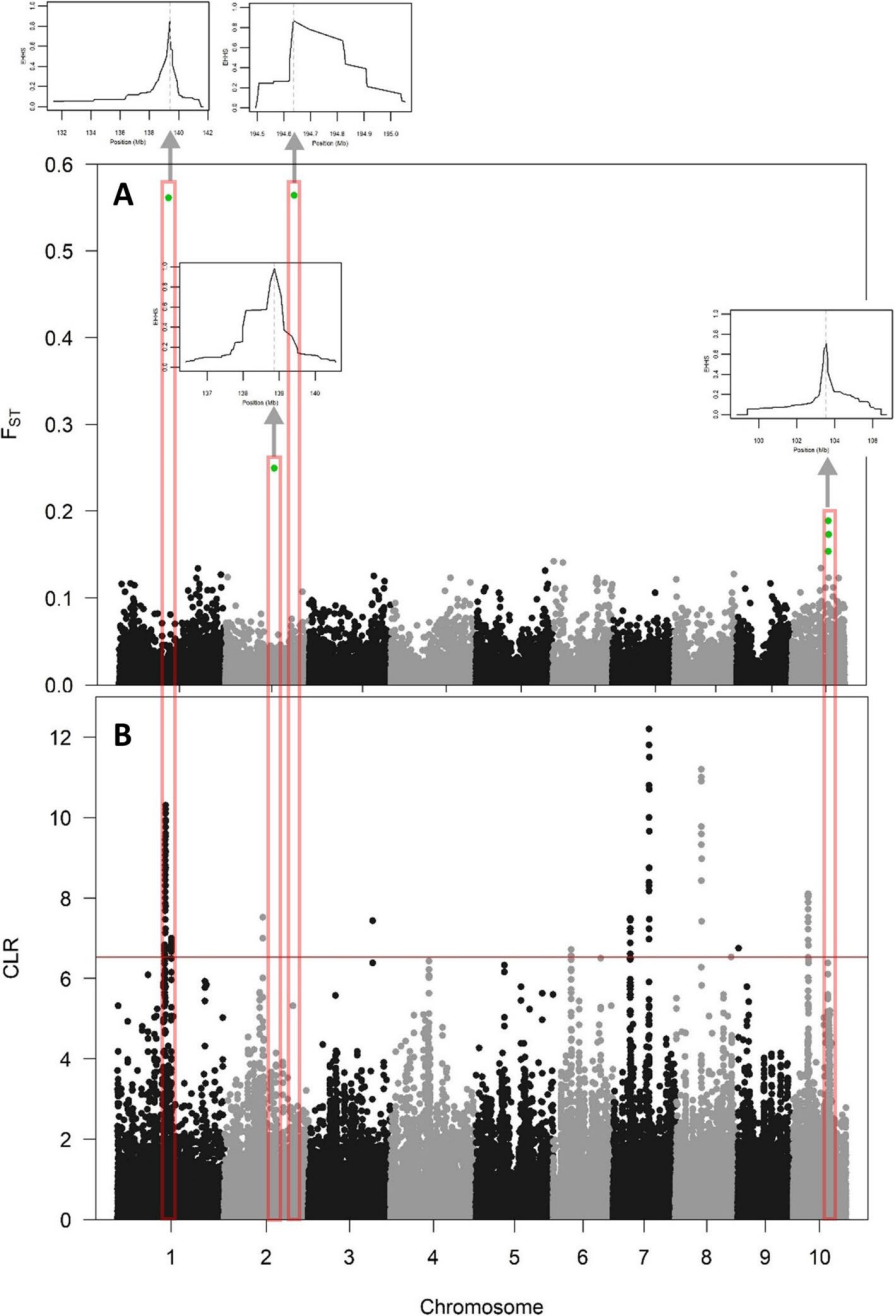
Genomewide fixation index (F_ST_) between 455 inbreds from the MRIZP-SEE subpanel and the rest of the inbreds from all three panels (**A**). Positions crossing an arbitrary F_ST_ threshold of 0.15 are shown in green with corresponding positional extended haplotype homozygosity per site (EHHS) scores (insets). EHHS insets show from left to right 10, 10, 0.6 and 8 Mbp. Red vertical bounding boxes denote overlapping regions with genomewide composite likelihood ratios (CLR) in the MRIZP-SEE subpanel (**B**). Boxes are arbitrary and do not represent exact physical positions. The red horizontal line represents a 0.1% CLR threshold of 6.530

Across all variants, a genomewide mean F_ST_ value of 0.0077 was observed between the 455 inbreds from the MRIZP-SEE subpanel and the remaining 519 inbreds from all three panels (519). However, in a sliding window analysis, four regions showed differentiation higher than an arbitrary threshold for fixation of 0.15 (Fig. 4A). Two regions on chromosomes 1 and 2 showed higher rates of fixation (0.561 and 0.564) compared to the other regions on chromosome 2 (0.249) and the one on chromosome 10 (0.189). All four detected regions were accompanied by higher than expected extended haplotype homozygosity scores (EHHS, in boxes). EHHS signals of selection were not further analyzed due to signals over many regions. Three of the detected regions with increased F_ST_ also showed corresponding increased values of CLR (> 5, Table 2). Regions on chromosome 1 between 141.626 and 141.627 Mbp fell within regions with significant CLR scores (Tables 2 and 3). Region on chromosome 2 at 200.527 showed a corresponding CLR value of 5.31 (not significant), although four Mbp apart. Regions with high F_ST_ on chromosome 10 showed several counterparts with CLR > 5 (not significant) and with locations between 101.655 and 106.472 Mbp.

**Table 2:** Summary of regions showing significant F_ST_ divergence

**Chr**	**Start (Mbp)**	**End (Mbp)**	**F**_**ST**_	**Candidate genes**
1	141.626	141.627	**0.561**	Palmitoyltransferase DHHC domain, Pollen allergen Ole e 1 family
2	143.373	143.374	**0.249**	Zm00001eb091930
2	200.527	200.528	**0.564**	Transcription factor TCP, Hydroxymethylglutaryl-coenzyme A synthase, Thiolase-like, Arsenical pump ATPase ArsA/GET3, Anion-transporting ATPase-like domain, P-loop containing nucleoside triphosphate hydrolase, XPG/Rad2 endonuclease, PIN-like domain superfamily, 5’-3’ exonuclease C-terminal domain superfamily, AP2/ERF domain, Multi antimicrobial extrusion protein
10	104.110	104.111	**0.189**	Peptidase C54, WD40/YVTN repeat-like-containing domain superfamily, Papain-like cysteine peptidase superfamily, Protein kinase domain, Bulb-type lectin domain superfamily, Armadillo, Pectate lyase, AmbAllergen,
10	104.607	104.608	**0.173**	Zinc finger RING-type, PA domain, Rhodanese-like domain,
10	105.032	105.033	**0.153**	Glycoside hydrolase superfamily, Galactose-binding-like domain superfamily

**Table 3:** Summary of genomic regions for the top 100 composite likelihood ratios (CLR) from SweeD scan along with the number of SNPs and number of genes per region

**Chr**	**Start (Mb)**	**End (Mb)**	**CLR**	**Length (Mb)**	**No. SNP**	**No. Genes**
1	115.14–116.63	152.28–154.8	6.53–10.3	35.9–39.65	411–457	218–249
1	117.14–147.89	147.16–159.21	6.66–9.94	7.79–36.42	149–407	74–223
1	117.14–119.04	147.16–153.56	6.8–9.94	28.72–36.42	271–407	163–223
1	143.63–147.89	155.68–159.21	6.66–7	7.79–15.58	149–286	74–133
2	106.27–106.27	110.65–110.65	7–7.52	4.38–4.38	66–66	43–43
3	181.65	181.91	7.43	0.26	32	7
6	49.3	54.46	6.72	5.16	30	41
7	45.57–45.93	52.43–52.91	6.61–7.49	6.5–7.35	103–113	60–64
7	98.86–100.8	103.97–106.23	6.97–12.2	3.17–7.37	56–140	28–76
8	70.46–72.98	74.44–76.86	7.42–11.21	1.46–6.4	38–152	35–102
9	2.88	3.26	6.75	0.37	18	5
10	36.23–37.54	49.07–50.58	6.82–8.1	11.53–14.35	105–137	69–85

Another approach used to detect selection in the 455 SEE inbred lines was the screening of changes in site frequency spectrum (SFS) of genomic regions compared to the neutral model, implemented in SweeD software [49]. The change compared to the neutral model was used to calculate the composite likelihood ratio (CLR) test of the regions with a shift in SFS. The top 0.1% hits from the composite likelihood ratio (CLR) test in SweeD software were considered significant, and 11 genomic regions were detected on chromosomes 1, 2, 3, 6, 7, 8, 9, and 10 (Fig. 4B).

TheBioMart analysis output a total of 722 genes harbored within the top 100 CLR regions with 883 putative products (Supplementary Table S4). Detected genomic regions contained from 18 to 457 SNPs and harbored 5 to 249 genes (Table 3). Only two regions were shorter than 1 Mbp on chromosomes 3 (260 kbp) and 9 (370 kbp), probably representing farther historical selection breaking apart the linkage disequilibrium, compared to the other regions spanning 1.46 to 39.65 Mbp.

### Ontology enrichment in positively selected genomic regions

The four regions showing divergence with values of fixation index (F_ST_) > 0.15 (Fig. 4) harbored 20 genes in total. Most of the detected genes were associated with transporter activities (Supplementary Table S3). Gene ontology enrichment analysis showed highly significant enrichment of molecular functions linked to passive transmembrane transporter activity (GO:0022803), more specifically, channel activity (GO:0015267). Another significantly enriched molecular function was inorganic solute uptake transmembrane transporter activity (GO:0015318).

**Fig. 5:**
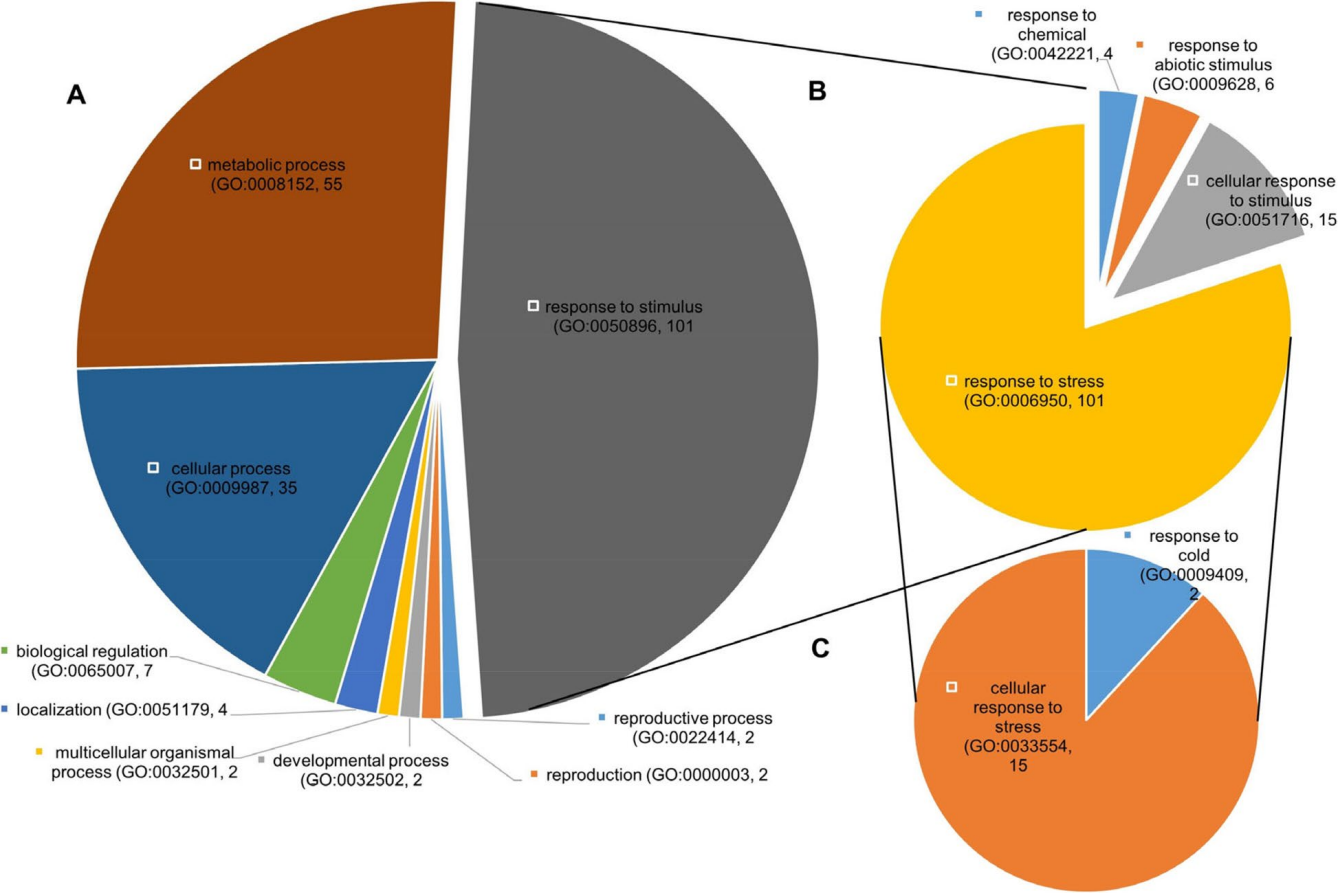
Significantly enriched biological processes (**A**) based on gene ontology (GO) of genes found in regions detected by SweeD procedure. The 101 genes associated with GO term Response to stimulus were further analyzed for enrichment (**B**) as well as the GO term response to stress (**C**)

Genes found in regions detected by SweeD (Supplementary Tables S6-S8) were also subjected to ontology enrichment analysis. Significant enrichment was detected for biological pathways (GO: 0008150), molecular functions (GO: 0003674), and cellular components (GO: 0005575). Enrichment for biological pathways GO showed that 101 of the 883 genes detected in regions showing signatures of selection, detected using SweeD methodology, were related to response to stimuli (Fig. 5A). This term was further analyzed and all of the 101 genes involved in *response to stimuli* were involved in biological pathway *response to stress* (Fig. 5B). Significant enrichment was also found in GO term *response to stress*, as enrichment of genes involved in response to specific stresses (Fig. 5C) and 15 genes were involved in the cellular response to stress, while two genes were involved in response to cold.

## Discussion

### Genetic structure of SEE genotyping panel

Unlike Western and Northwestern Europe, in which flint x temperate dent combinations are mostly utilized for single cross hybrids [14, 53], the climatological conditions of the Southeast and East Central Europe show a strong resemblance to those of the US Corn Belt and Midwest, where a significant proportion of U.S. corn is grown [54]. In the US, the Northern Flint and Southern Dent races had been crossed to form the Corn Belt Dent race in the 1800s. Because the Corn Belt Dent and Southern Dent races were so much more productive than the Northern Flints, their role established in this region by the end of the 1800s under the influence of American Corn Shows [55], leaving no strong evidence of Northern Flints playing an important role besides the historical admixture between the Southern Dents and the Northern Flints [56]. Moreover, the germplasm fluctuation was supported by a substantial North-American diaspora of Southeast European countries [57] and their long maritime culture. The utilization of maize landraces and OPVs for hybrid breeding in Southeast Europe was very popular within the early maize-breeding community of this region [8], resulting in the first registered double-cross, three-way cross, and single cross hybrids with original and semi-original formulas during the 1960s [18]. However, the registration of public, single cross hybrid B73xMo17 by W.A. Russell, L.H. Penny, and A.R. Hallauer at Iowa State University in 1974 marked a milestone in maize breeding and commercialization, as the hybrid surpassed all its predecessors in agronomic performance and stability [58], and the parental inbreds B73 and Mo17 along with B84, N7A, Pa91, H102, A632, etc. became standard sources of favorable alleles [59, 60].

The MRIZP panel described in this work consists mostly of inbred lines stored in the MRIZP Gene Bank prior to 1980, which were used for commercial breeding and research activities, with few exceptions, mostly representing the pre-B73xMo17 era of breeding. This is also visible in Fig. 1A, where only a small share of inbreds shows pure pedigrees of B73/B14, B37, and Wf9/Oh07 groups, whereas most of the inbreds are admixed. Admixture represents a form of genomic adaptation to environmental deviations [61], which was already utilized in adaptation of maize to European climates [62]. However, the underrepresentation of pure European flint materials in the MRIZP panel and the high number of admixed inbreds bearing the blends of various 20^th^ century genomes indicates possible germplasm swaps in Southeast Europe. The swapping of the original flint materials in this region already happened before, when the local European flint materials, resembling the Caribbean and the South-American landraces, were swapped by US dent OPVs [8, 55]. Unlike Western Europe, where the dent xflint heterotic pattern is exploited [53], dent x dent formulas are preferred in Southeast Europe where environments represent the US Corn Belt agro-ecological conditions more closely [1, 5].

The analysis of only 455 inbreds collected from Southeast Europe, i.e. MRIZP-SEE subpanel (Figs. 1B and 3) also supports this speculation, as the pure inbreds (Q > 0.7) from all three identified groups show Q of no more than 0.72 in any of the seven groups identified in K = 7 analysis of the entire panel (Fig. 1A). Most appealingly, the SEE sub-panel almost completely lacks Iodent germplasm which is the most recently established as a separate heterotic group [25]. The other two panels from TUM and DROPS [14, 37], contain Iodent inbreds from sources with expired Plant Variety Certificates (ex-PVP) and more recent breeding activities. The lack of Iodent in the SEE subset (Fig. 2A and B) emphasizes the historical context of the panel. Iodent is a germplasm group discovered and initially almost exclusively exploited by Pioneer Hi-Bred [63] from the 1980s until the expiration of the PVP certificates, followed by the development of several accessions by Forest Troyer at Dekalb [17]. The closest grouping of inbreds to the Iodent group in our study with Q ~ 0.7 possibly representing inbreds developed from early OPVs originating from the Reid Yellow dent breeding program, from which the Iodent group also originates [25]. Analysis of the spatial distribution of different genetic groups in our study (Supplementary Fig. 3) showed a high amount of genomic diversity accounted by Flint materials and Iodents in France and Germany, while at the Eastward regions, the prevalence of Wf9/Oh07, B37, and A374 materials was observed (Supplementary Fig. 3). Moreover, a shocking 91.1% of the SEE materials showed Q > 0.1 in the A374 group. The A374 inbred was developed at Holbert in Illinois by Reid Yellow Dent [64], which was the most diverse US historical germplasm sources [28]. Other significant groups substantially represented in the SEE panel, such as B37, Wf9, and European flint, represent heterotic partners to the A374 group [65].

### Signatures of selection and pathway enrichment in Southeast Europe

To further analyze the genomic implications of population expansion observed in the MRIZP panel, genomewide scans for signatures of selection were carried out. To facilitate the robust search for signatures of selection and avoid the detection of false positives in data generated by SNP-array suffering from ascertainment bias of polymorphism states, a stringent SNP pruning mitigation strategy was applied, as suggested by [45]. The scan for sweeps was based on a SweepFinder method [34] implemented for large genomewide SNP data in SweeD software [49]. The output statistics are based on a linkage disequilibrium of the selected variant disturbing its surrounding regions, creating shifts in the site frequency spectrum (SFS). SweeD calculates the SFS of the genomewide neutral model and compares its likelihood to the likelihood of SFS shifts in the assessed window to output composite likelihood ratio (CLR) statistics.

Detection of the long physical regions with signs of selection can be caused by several factors. Inbreeding is known to shift the SFS as the number of effective recombination events are reduced [66], producing long regions with significant signals. Another factor affecting the length of sweep regions is the strength of the allelic effect of the selected region and the speed at which it spreads through the population, i.e. if the spreading of the variant is faster than the recombination, linkage disequilibrium will not have enough time to diminish [14]. Also, long regions can be caused by the effect known as “soft shoulder”, flanking the regions with hard sweeps [67]. The potential remedy for such occurrences is to screen a broader region for sweep signals, rather than to aim to classify the sweep as “hard” or “soft”. Our speculation is that the long regions with increased CLR detected in our study (Fig. 4, Table 3) represent recent selection in the materials from the SEE panel (except signals on chromosomes 3 and 9). This is in accordance with the time that passed from the introduction of new US Corn Belt materials in Southeast Europe during the 1950s [22] and the first cycle of registered inbreds during the 1960s [18] This is also corroborated by the timing,, considering that most of the inbreds of the SEE panel were stored in MRIZP Genbank during the 1960s and 1970s, leaving time for only a single cycle of selection. In line with this speculation is also the finding of very low levels of F_ST_ divergence between 519 inbreds from all three genotyping panels and 455 inbreds collected from Southeast Europe, except at four regions (Fig. 4A). Selection of contrast population for fixation followed two heuristics. First was that the inbreds present in contrast population from TUM and DROPS panels were mostly founder lines of each of the known heterotic groups in US [63] as well as in Europe [68]. The other heuristic relied on the assumption that the inbreds from other countries that were stored to MRIZP Gene bank were probably used as donors in SEE breeding programs. There are several shortcomings of such approach, as the target population of 455 SEE inbreds clearly represents the population expansion [69]. Furthermore, potential rare variants [70] might also cause artefacts in the analysis, however, due to ascertainment towards existing variants in array based genotyping and the applied mitigation strategy in our study, this issue might not be relevant to our study. Both target and contrast populations in our study were structured, which might increase the fixation time, however, increasing the effective rate of evolution or adaptation [71].

To further confirm population divergence at detected loci, regions with high F_ST_ were also screened for EHHS. Haplotype tests also suffer from several limitations and rely on haplotype lengths around focal positions and are usually amplified by inbreeding [72]. Accordingly, the results of EHHS for all regions were not subject to further analysis. In joint analysis of CLR and F_ST_, low resemblance between scores was observed. There is generally low resemblance between F_ST_ and SFS-based methods for screening selection due to their different assumptions. Namely, F_ST_ represents population divergence through fixation of alleles, while SFS-based methods aim for more subtle changes in allelic composition of broader genomic regions [73]. Also, flanking regions to region under hard selective sweep [66, 67] might shift positions of the selective signals.

Regions showing signatures of selection were scanned for candidate genes which were subjected to gene ontology (GO) enrichment analysis [52] for three main terms: *molecular function, biological process,* and *cellular component*. GO terms related to passive transmembrane transporter activity, channel activity, and inorganic solute uptake transmembrane transporter activity of GO molecular function were significantly enriched in both CLR and F_ST_ based analysis (Supplementary Tables S5 and S7). During the 1960s, a period often called “the Green revolution”, agronomy was redefined in general with the spread of the practice of mineral fertilization, advances in plant breeding theory and practices, and mass availability of agricultural machinery [74]. We speculate that the breeding programs in Southeast Europe needed to adapt to standards of stress tolerance and nutrient use efficiency imposed by novel US developments, which were subsequently admixed to local materials and utilized as sources of favorable allelic diversity. For example, in the GO analysis of high CLR regions (Fig. 4B, Table 3), the molecular function enrichment showed a highly significant (*p* = 4.25^–28^) overrepresentation of UDP-glucosyltransferase activity.

Recent research suggests that in rice, the activity of UDP-glucosyltransferase redirects metabolic flux and directly affects plant stress tolerance and grain size [75]. Moreover, another significantly enriched molecular function (*p* = 2.26^–13^) was passive transmembrane transporter activity, more specifically, channel activity. Passive transport is any transport that occurs due to the concentration, pressure, or electric potential gradient, and it represents a way for plants to absorb nutrients and water [76]. Also, transport facilitated by channels is the main process of communication between cells [77]. The analysis of GO-term biological processes showed two highly significant enrichments. Firstly, the significantly enriched processes nitrogen compound mechanism process (GO:0006807) and oligosaccharide biosynthetic process (GO:0009312) appear to be the key adaptational mechanisms to low N conditions [78], probably indicating selection for nitrogen use efficiency in N-depleted environments. Secondly, highly significant enrichment (*p* = 1.54^–57^) of process response to stress (GO:0006950) with 101 involved genes further significantly enriched for cellular response to stress and response to cold (Fig. 5) possibly marks SEE genotyping panel and MRIZP Gene Bank as a top-tier resource of adaptational alleles for changing climate. Interestingly, a similar study in European landraces showed significant enrichment in adaptational regions under selection [62]. However, the GO terms did not overlap with the results of our study (except for functions regarding vesicles and transport), possibly due to the presence of more modern breeding material with other targeted outcomes of selection compared to historical OPVs.

## Supplementary Information

**Additional file 1: Supplementary table S1.** Information about the full set of 974 inbreds used, along with membership probabilities from the Admixture analysis. **Supplementary table S2.** Information about the SEE subpanel of inbreds used, along with membership probabilities from the Admixture analysis. **Supplementary table S3.** Results of BioMart database mining for genes linked to regions with significant fixation index. **Supplementary table S4.** Results of BioMart database mining for genes linked to regions with significant fixation index. **Supplementary table S5.** Results of gene ontology (GO) enrichment analysis for three main term molecular function for genes linked to regions with significant fixation index. **Supplementary table S6.** Results of gene ontology (GO) enrichment analysis for three main term biological procesess for genes linked to regions with significant CLR. **Supplementary table S7.** Results of gene ontology (GO) enrichment analysis for three main term molecular function for genes linked to regions with significant CLR. **Supplementary table S8.** Results of gene ontology (GO) enrichment analysis for three main term cellular component for genes linked to regions with significant CLR.

**Additional file 2: Supplementary figure 1.** Structure Selector output converging at K = 7 for all four examined parameters in the full dataset of 974 inbreds. **Supplementary figure 2.** Structure Selector output converging at K = 2 for all four examined parameters in the MRIZP-SEE subpanel of 455 inbreds. **Supplementary figure 3.** Average admixture results assigned to every putative country of origin from the full dataset. K1 represents European flint, K2 B73/B14, K3 Lancaster, K4 B37, K5 Wf9/Oh, K6 A374 and K7 Iodent.

## Data Availability

All data and plant material used to draw conclusions presented in this manuscript is available upon reasonable request to the corresponding author.

## References

[1] Leff B, Ramankutty N, Foley JA. Geographic distribution of major crops across the world. Glob Biogeochem Cycles. 2004;18:1–27.

[2] USDA. United States Department of Agriculture National Agricultural Statistics Service. 2020.

[3] Republic of Serbia. Statistical Office of the Republic of Serbia. 2020.

[4] Eurostat. Agricultural production - crops. 2019.

[5] Jambrović A, Mazur M, Radan Z, Zdunić Z, Ledenčan T, Brkić A, et al. Array-based genotyping and genetic dissimilarity analysis of a set of maize inbred lines belonging to different heterotic groups. 2014. Genetika. 10.2298/GENSR1402343J.

[6] Şuteu D, Bǎcilǎ I, Haş V, Haş I, Miclǎuş M (2013). Romanian maize (Zea mays) inbred lines as a source of genetic diversity in SE Europe, and their potential in future breeding efforts. PLoS One.

[7] Andjelkovic V, Nikolic A, Kovacevic D, Mladenovic-Drinic S, Kravic N, Babic V (2018). Conserving maize in gene banks: changes in genetic diversity revealed by morphological and SSR markers. Chil J Agric Res.

[8] Leng ER, Tavčar A, Trifunovič V (1962). Maize of southeastern Europe and its potential value in breeding programs elsewhere. Euphytica.

[9] Vančetović J, Mladenović Drinić S, Babić M, Ignjatović-Micić D, Andelković V (2010). Maize genebank collections as potentially valuable breeding material. Genetika.

[10] Gouesnard B, Negro S, Laffray A, Glaubitz J, Melchinger A, Revilla P (2017). Genotyping-by-sequencing highlights original diversity patterns within a European collection of 1191 maize flint lines, as compared to the maize USDA genebank. Theor Appl Genet.

[11] Planchenault D, Mounolou JC (2011). Evolutions and stakes of genetic resources management. Comptes Rendus - Biol.

[12] Ortiz R, Taba S, Chávez Tovar VH, Mezzalama M, Xu Y, Yan J (2010). Conserving and enhancing maize genetic resources as global public goods-a perspective from CIMMYT. Crop Sci.

[13] Sood S, Flint-Garcia S, Willcox MC, Holland JB. Mining natural variation for maize improvement: selection on phenotypes and genes. In: Tuberosa R, Graner A, Frison E, editors. Genomics of plant genetic resources: volume 1. Managing, sequencing and mining genetic resources. Dordrecht: Springer; 2014. p. 615–49.

[14] Unterseer S, Pophaly SD, Peis R, Westermeier P, Mayer M, Seidel MA (2016). A comprehensive study of the genomic differentiation between temperate Dent and Flint maize. Genome Biol.

[15] Hölker AC, Mayer M, Presterl T, Bolduan T, Bauer E, Ordas B (2019). European maize landraces made accessible for plant breeding and genome-based studies. Theor Appl Genet.

[16] Allier A, Teyssèdre S, Lehermeier C, Moreau L, Charcosset A (2020). Optimized breeding strategies to harness genetic resources with different performance levels. BMC Genomics.

[17] Lee EA, Tracy WF. Modern maize breeding. In: Bennetzen J, Hake S, editors. Handbook of maize: genetics and genomics. New York: Springer Science+Business Media, LLC; 2009. p. 151–60.

[18] Rojc M, Parlov D, Stastny K, Kozić Z, Vragolović A (1983). Dostignuća u selekciji linija i hibrida kukuruza u SR Hrvatskoj - in Croatian. Agron Glas.

[19] Tenaillon MI, Charcosset A (2011). A European perspective on maize history. Comptes Rendus - Biol.

[20] Brkić I, Parlov D, Kozumplik V. Maize seed production in Croatia. In: Ruckenbauer P, editor. Bericht über die 54. Tagung 2003 der Vereinigung der Pflanzenzüchter und Saatgutkaufleute Österreichs. 2003. p. 1–5.

[21] Hadi G, Pinter J, Marton C. The first 30 years of hybrid maize in Hungary. In: 60 years of Hungarian hybrid maize. Budapest: Pannonian Plant Biotechnology Association; 2013. p. 112–6.

[22] Tavčar A (1955). Methods of hybrid maize production in Yugoslavia (in Croatian). Agron Glas.

[23] White MR, Mikel MA, de Leon N, Kaeppler SM (2020). Diversity and heterotic patterns in North American proprietary dent maize germplasm. Crop Sci.

[24] Mikel MA (2011). Genetic composition of contemporary U.S. commercial dent corn germplasm. Crop Sci.

[25] Troyer AF (1999). Background of U.S. hybrid corn. Crop Sci.

[26] Troyer AF (2004). Background of U.S. hybrid corn II: breeding, climate, and food. Crop Sci.

[27] Troyer AF (2009). Development of hybrid corn and the seed corn industry. Handb Maize Genet Genomics.

[28] Lu H, Bernardo R (2001). Molecular marker diversity among current and historical maize inbreds. Theor Appl Genet.

[29] FAO/IHS Markit Agribusiness Consulting. Analysis on sales and profitability within the seed sector. 2019.

[30] Reif JC, Hamrit S, Heckenberger M, Schipprack W, Maurer HP, Bohn M (2005). Trends in genetic diversity among European maize cultivars and their parental components during the past 50 years. Theor Appl Genet.

[31] Bouchet S, Servin B, Bertin P, Madur D, Combes V, Dumas F (2013). Adaptation of maize to temperate climates: mid-density genome-wide association genetics and diversity patterns reveal key genomic regions, with a major contribution of the Vgt2 (ZCN8) locus. PLoS One.

[32] Romero Navarro JA, Willcox M, Burgueño J, Romay C, Swarts K, Trachsel S (2017). A study of allelic diversity underlying flowering-time adaptation in maize landraces. Nat Genet.

[33] Wegary D, Teklewold A, Prasanna BM, Ertiro BT, Alachiotis N, Negera D (2019). Molecular diversity and selective sweeps in maize inbred lines adapted to African highlands. Sci Rep.

[34] Nielsen R, Williamson S, Kim Y, Hubisz MJ, Clark AG, Bustamante C (2005). Genomic scans for selective sweeps using SNP data. Genome Res.

[35] Harris AM, Garud NR, Degiorgio M (2018). Detection and classification of hard and soft sweeps from unphased genotypes by multilocus genotype identity. Genetics.

[36] Unterseer S, Bauer E, Haberer G, Seidel M, Knaak C, Ouzunova M (2014). A powerful tool for genome analysis in maize: development and evaluation of the high density 600 k SNP genotyping array. BMC Genomics.

[37] Millet E, Welcker C, Kruijer W, Negro S, Nicolas S, Praud S (2016). Genome-wide analysis of yield in Europe: allelic effects as functions of drought and heat scenarios. Plant Physiol.

[38] Negro SS, Millet EJ, Madur D, Bauland C, Combes V, Welcker C (2019). Genotyping-by-sequencing and SNP-arrays are complementary for detecting quantitative trait loci by tagging different haplotypes in association studies. BMC Plant Biol.

[39] Bradbury PJ, Zhang Z, Kroon DE, Casstevens TM, Ramdoss Y, Buckler ES (2007). TASSEL: software for association mapping of complex traits in diverse samples. Bioinformatics.

[40] Money D, Gardner K, Migicovsky Z, Schwaninger H, Zhong GY, Myles S (2015). LinkImpute: Fast and accurate genotype imputation for nonmodel organisms. G3.

[41] Alexander DH, Lange K (2011). Enhancements to the ADMIXTURE algorithm for individual ancestry estimation. BMC Bioinformatics.

[42] Puechmaille SJ (2016). The program structure does not reliably recover the correct population structure when sampling is uneven: subsampling and new estimators alleviate the problem. Mol Ecol Resour.

[43] Li YL, Liu JX (2018). StructureSelector: a web-based software to select and visualize the optimal number of clusters using multiple methods. Mol Ecol Resour.

[44] Ganal MW, Durstewitz G, Polley A, Bérard A, Buckler ES, Charcosset A (2011). A large maize (zea mays L.) SNP genotyping array: development and germplasm genotyping, and genetic mapping to compare with the B73 reference genome. PLoS One.

[45] Malomane DK, Reimer C, Weigend S, Weigend A, Sharifi AR, Simianer H (2018). Efficiency of different strategies to mitigate ascertainment bias when using SNP panels in diversity studies. BMC Genomics.

[46] Purcell S, Neale B, Todd-Brown K, Thomas L, Ferreira MAR, Bender D (2007). PLINK: a tool set for whole-genome association and population-based linkage analyses. Am J Hum Genet.

[47] Weir BS, Cockerham CC (1984). Estimating F-statistics for the analysis of population structure. Evolution (N Y).

[48] Gautier M, Klassmann A, Vitalis R (2017). rehh 2.0: a reimplementation of the R package rehh to detect positive selection from haplotype structure. Mol Ecol Resour.

[49] Pavlidis P, Živković D, Stamatakis A, Alachiotis N (2013). SweeD: likelihood-based detection of selective sweeps in thousands of genomes. Mol Biol Evol.

[50] Howe KL, Contreras-Moreira B, De Silva N, Maslen G, Akanni W, Allen J (2020). Ensembl Genomes 2020-enabling non-vertebrate genomic research. Nucleic Acids Res.

[51] Klassmann A, Gautier M (2022). Detecting selection using extended haplotype homozygosity (EHH)-based statistics in unphased or unpolarized data. PLoS One.

[52] Mi H, Muruganujan A, Thomas PD (2013). PANTHER in 2013: modeling the evolution of gene function, and other gene attributes, in the context of phylogenetic trees. Nucleic Acids Res.

[53] Cartea ME, Revilla P, Butrón A, Malvar RA, Ordás A (1999). Do second cycle maize inbreds preserve the European flint heterotic group?. Crop Sci.

[54] Green TR, Kipka H, David O, McMaster GS (2018). Where is the USA Corn Belt, and how is it changing?. Sci Total Environ.

[55] Hadi G (2006). Genetic basis of maize production in Eastern Central Europe between 1610 and 2005: review. Cereal Res Commun.

[56] Anderson E, Brown WL, Gowen JW (1952). Origin of Corn Belt maize and its genetic significance. Heterosis.

[57] Cohen R. The Cambridge survey of world migration. Cambridge, UK: Cambridge University Press; 1995.

[58] Smith C, Betran J, Runge EC. Corn: origin, history, technology, and production. New Jersey: Wiley; 2004.

[59] Mišević D (1989). Evaluation of three test statistics used to identify maize inbred lines with new favorable alleles not present in elite single cross. Theor Appl Genet.

[60] Troyer AF (2006). Adaptedness and heterosis in corn and mule hybrids. Crop Sci.

[61] Calfee E, Gates D, Lorant A, Perkins MT, Coop G, Ross-Ibarra J (2021). Selective sorting of ancestral introgression in maize and teosinte along an elevational cline. PLoS Genet.

[62] Brandenburg J, Mary-huard T, Rigaill G, Hearne SJ, Joets J, Charcosset A, et al. Independent introductions and admixtures have contributed to adaptation of European maize and its American counterparts. PLoS Genet. 2017;17:e1006666. 10.1371/journal.pgen.1006666 PMC5373671 28301472

[63] Mikel MA, Dudley JW (2006). Evolution of North American dent corn from public to proprietary germplasm. Crop Sci.

[64] Hayes HK, Rinke EH, Tsiang YS. Experimental study of convergent improvement and backcrossing in corn. Minnesota: University of Minnesota, Minnesota Agricultural Experiment Station; 1946.

[65] Dubreuil P, Dufour P, Krejci E, Causse M, De Vienne D, Gallais A (1996). Organization of RFLP diversity among inbred lines of maize representing the most significant heterotic groups. Crop Sci.

[66] Hartfield M, Bataillon T (2020). Selective sweeps under dominance and inbreeding. G3.

[67] Schrider DR, Mendes FK, Hahn MW, Kern AD (2015). Soft shoulders ahead: spurious signatures of soft and partial selective sweeps result from linked hard sweeps. Genetics.

[68] Rebourg C, Chastanet M, Gouesnard B, Welcker C, Dubreuil P, Charcosset A (2003). Maize introduction into Europe: the history reviewed in the light of molecular data. Theor Appl Genet.

[69] Kitada S, Nakamichi R, Kishino H (2021). Understanding population structure in an evolutionary context: Population-specific FST and pairwise FST. G3.

[70] Bhatia G, Patterson N, Sankararaman S, Price AL (2013). Estimating and interpreting FST: the impact of rare variants. Genome Res.

[71] Tkadlec J, Pavlogiannis A, Chatterjee K, Nowak MA (2019). Population structure determines the tradeoff between fixation probability and fixation time. Commun Biol.

[72] Hartfield M, Bataillon T, Glémin S (2017). The evolutionary interplay between adaptation and self-fertilization. Trends Genet.

[73] Ma Y, Ding X, Qanbari S, Weigend S, Zhang Q, Simianer H (2015). Properties of different selection signature statistics and a new strategy for combining them. Heredity (Edinb).

[74] Hazell PBR. The Asian Green Revolution, vol 911. Washington, DC: International Food Policy Research Institute; 2009. p. 1–31

[75] Dong NQ, Sun Y, Guo T, Shi CL, Zhang YM, Kan Y (2020). UDP-glucosyltransferase regulates grain size and abiotic stress tolerance associated with metabolic flux redirection in rice. Nat Commun.

[76] Jarzyniak KM, Jasiński M (2014). Membrane transporters and drought resistance - a complex issue. Front Plant Sci.

[77] Tomkins M, Hughes N, Morris RJ. An update on passive transport in and out of plant cells. Plant Physiol. 2021;187:1973–84. 10.1093/plphys/kiab406 PMC8644452 35235675

[78] Schlüter U, Colmsee C, Scholz U, Bräutigam A, Weber APM, Zellerhoff N (2013). Adaptation of maize source leaf metabolism to stress related disturbances in carbon, nitrogen and phosphorus balance. BMC Genomics.

