# Supplementary material for: Genetic diversity and selection signatures in a gene bank panel of maize inbred lines from Southeast Europe compared with two West European panels
Source: BMC Plant Biol. 2023 Jun 14;23:315. doi: 10.1186/s12870-023-04336-2 (PMC10265872; doi:10.1186/s12870-023-04336-2)
Supplement: Supplementary file 2 — Additional file 2: Supplementary figure 1. Structure Selector output converging at K = 7 for all four examined parameters in the full dataset of 974 inbreds. Supplementary figure 2. Structure Selector output converging at K = 2 for all four examined parameters in the MRIZP-SEE subpanel of 455 inbreds. Supplementary figure 3. Average admixture results assigned to every putative country of origin from the full dataset. K1 represents European flint, K2 B73/B14, K3 Lancaster, K4 B37, K5 Wf9/Oh, K6 A374 and K7 Iodent. [file 12870_2023_4336_MOESM2_ESM.docx]

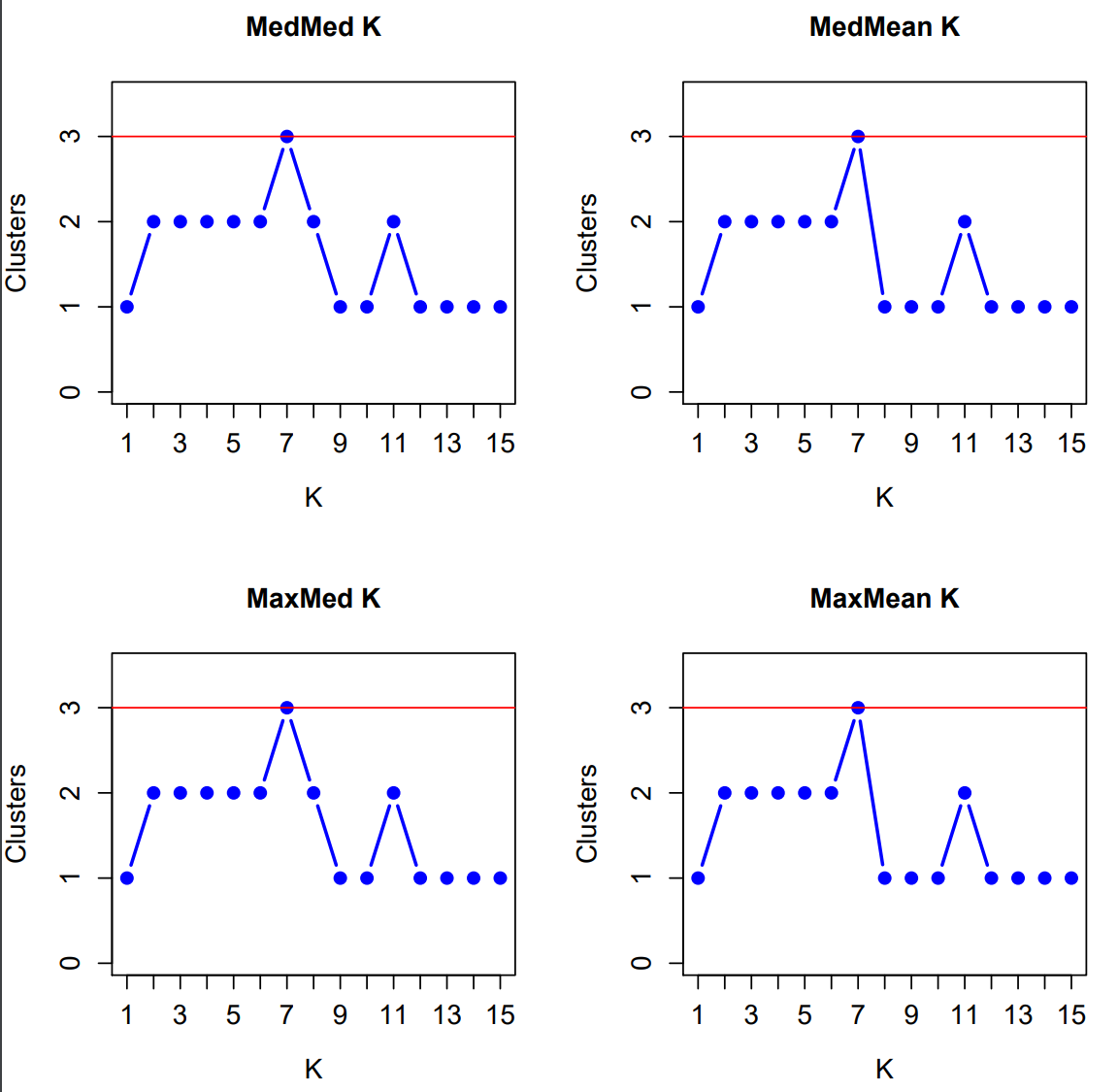


Supplementary figure 1. Structure Selector output converging at K=7 for all four examined parameters in the full dataset of 974 inbreds


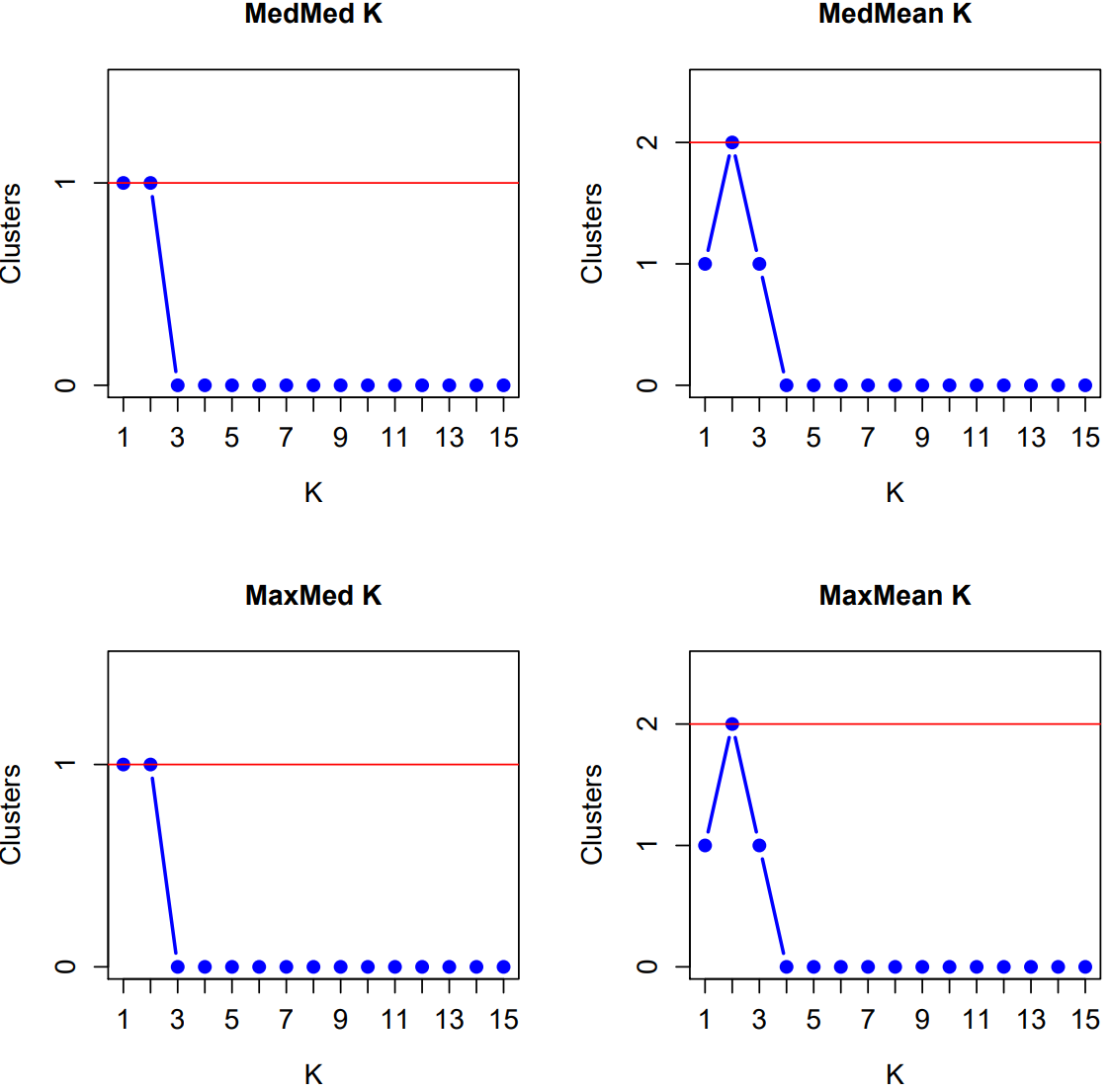


Supplementary figure 2. Structure Selector output converging at K=2 for all four examined parameters in the MRIZP-SEE subpanel of 455 inbreds


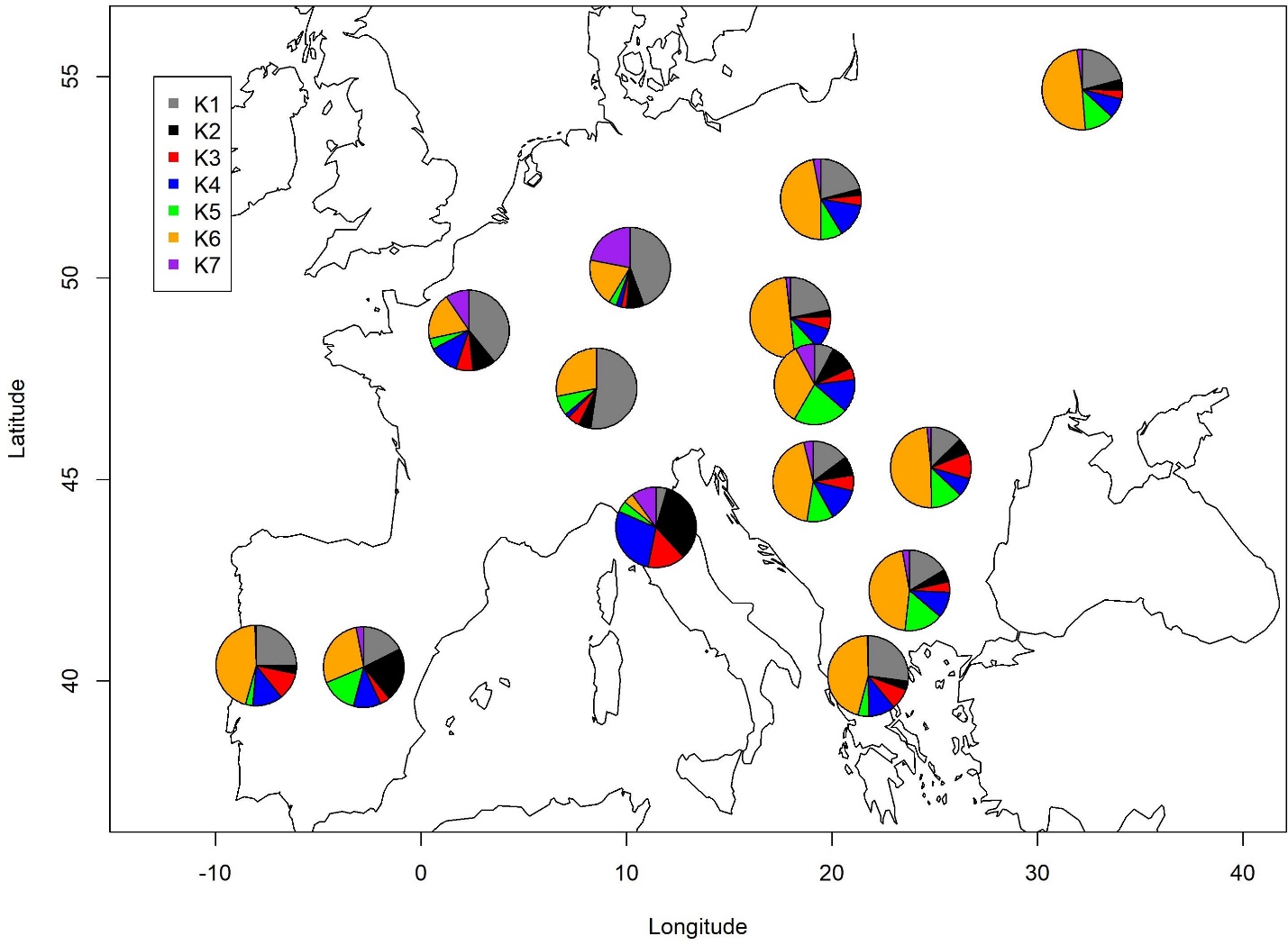


Supplementary figure 3. Average admixture results assigned to every putative country of origin from the full dataset. K1 represents European flint, K2 B73/B14, K3 Lancaster, K4 B37, K5 Wf9/Oh, K6 A374 and K7 Iodent
